# CA-XTree: Age Estimation of Grouped Gradient Regression Tree with Local Channel Attention

**DOI:** 10.1155/2022/4155461

**Published:** 2022-05-28

**Authors:** Xiaoding Lu, Zhengyou Wang, Yanhui Xia, Shanna Zhuang

**Affiliations:** ^1^School of Information Science and Technology, Shijiazhuang Tiedao University, Shijiazhuang, China; ^2^Hebei Key Laboratory for Electromagnetic Environmental Effects and Information Processing, Shijiazhuang 050043, China

## Abstract

Face age estimation has been widely used in video surveillance, human-computer interaction, market analysis, image processing analysis, and many fields. There are several problems that need to be solved in image-based face age estimation: (1) redundant information of age characteristics; (2) limitations of age estimation methods in solving age estimation problems; (3) the performance of age estimation models being also affected by gender factors. This paper proposes CA-XTree network. Firstly, features are extracted through the convolution layer and then combined with the local channel attention module to strengthen the ability of age feature information interaction between different channels. Secondly, extracted features are inputted into the recommendation score function to obtain the recommendation score, by combining the recommendation score with the gradient ascending regression tree. The lifting tree processed loss function is the mean square loss function, and the final age value is obtained by the leaf node. This paper improves state of the art for image classification on MORPH and CACD datasets. The advantage of our model is that it is easy to implement and has no excess memory overhead. In the age dataset CACD, the mean absolute error (MAE) has reached 4.535 and cumulative score (CS) has reached 63.53%, respectively.

## 1. Introduction

With the arrival of the information age and the wide application of various intelligent devices, computer vision technology has become a research hotspot. Age estimation, it provides a picture that automatically identifies the true age of the person in the image [1]. Using this algorithm to estimate age is a difficult task [2]. In recent years, great progress has been made in face recognition, but there are still many challenges in the cross-age recognition and retrieval. First of all, the face has different manifestations at different ages. Age is related to facial biological characteristics in different periods (for example, facial bone growth in children, facial wrinkles in adults). Secondly, age estimation is also affected by external factors such as illumination, posture, and expression. In addition, for individuals of different races [3], even if one's age is the same as the other, the skin color will age to varying degrees.

At present, there are many difficulties in the field of age estimation. Firstly, due to the spatial heterogeneity of face features, the prediction of real age estimation has always been a nonlinear mapping function. People of the same age have great differences in appearance, and faces have different manifestations at different ages. Secondly, due to insufficient training data and unclear labels, there is too much background information in the image, the accuracy of age estimation uses limited samples [4], and fuzzy labels are affected. Finally, in shopping markets, advertising campaign show to collect customer information and make relevant evaluations according to customers' age and gender, so as to realize targeted product recommendation services. From a biological point of view, the changes of facial contour and skin texture are affected by many factors such as living environment, race, and genetic differences, resulting in a nonstationary random process of aging pattern. Regression problem of nonstationary learning [5] is very difficult because it does not fit the training process. In addition, most face databases are under certain circumstances, such as on the Internet. In the real scene, due to the background, facial expression, and illumination [6] and partial shade, these [7] will limit the generalization ability of the model.

In the field of image classification, deep convolution neural network model mainly uses supervised learning to classify images, but it is limited by the quality and scale of dataset. The depth network is integrated in an end-to-end multilayer way. The depth of the network can enrich the “hierarchy” of features. Deep features have large acceptance domain and rich semantic information. Deep features are robust to the changes of object attitude, occlusion, and local deformation, but robust to geometric details [8] which are lost due to the reduction of resolution. The extraction of shallow features contains some of its details, and with the deepening of layers, the geometric details of extracted features may completely disappear [9, 10]. Recent evidence shows that network depth is crucial to improve accuracy and has achieved leading results on challenging ImageNet datasets [11] and recent evidence shows that network depth is crucial to improve accuracy and has achieved leading results on challenging ImageNet datasets.

Deep learning is a new research direction in the field of machine learning, and the representative model is deep convolutional neural network (CNN). With the development of convolutional neural network (CNN), designing residual network [12] is a breakthrough that has an impact on CNN. Based on the “Black box” phenomenon, the depth of the convolution of the neural network has been plagued by researchers for convolution neural network decision-making process and cannot intuitively describe network in what to do, such as what features were extracted from each layer, According to Chen et al. [1] the residual network cannot perform strict mathematical analysis, causing the remaining loss function of network learning process visualization. The visualization proves that the residual learning optimization problem is easier. Decision tree model (DT) [13] has a good model interpreter, which can provide good model interpretation. Deep decision tree combines features extracted from deep convolutional neural network with decision tree to improve model accuracy and increase model interpretability. At the same time, because it is combined with decision tree, it can also provide basis for model judgment clearly and step by step. In recent years, soft splitting functions [14–16] have been used to extend traditional decision trees to deep and deep path decision forests, so that decision trees have deep representation learning ability.

Face age in biology is the most intuitive expression in the human face, and face facial aging is a nonsmooth process, mainly divided into three parts, respectively, the juvenile period, middle age, and old age. At present, how to estimate the age of face image is particularly important in age estimation research. Age estimation method is mainly divided into multiple classification method and regression method, using relative order, between age and age tags in [8, 16]. Through the comparison between different individuals, by comparing the results with the change of the age difference, change the present *s* type curve. The CA-NeXt network is composed of local channel attention modules and grouped volumes.

**Figure 1:**
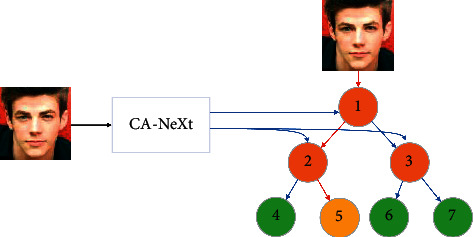
CA-XTree network structure diagram, which is composed of local channel attention ResNet network and regression tree classification.

We propose a simple architecture, as shown in Figure 1. Regression tree age classification network based on local channel attention, by not dimension reduction of global average pooling level channel, implements each age's characteristics of the channel and the interaction between *K* led 7 characteristics, module is made up of a one-dimensional fast convolution [17], the size of the one-dimensional convolution *K* represents the local channel number and the interactive age characteristics of the book, the last type is output in the whole connection layer, and the output results were input into the Softmax function to calculate the scoring function. The scoring function results were input into the regression tree, and the final age estimation results were obtained through the regression tree model. In general, the main contributions of this paper are as follows:

1. We design the residual network with regression tree (RT) to train the CA-XTree structure through tree supervision loss. High experimental results are obtained.
2. Lightweight channel focus modules are added to the remaining modules to effectively capture information between channels, to realize the information interaction between channels with the characteristics of the times.
3. We split the convolution using a compromise between grouping convolution and choosing ordinary convolution and depth, and the number of channels generated for each branch map is *n* (*n* > 1). The balance between the two strategies is achieved by controlling the number of organizations, with fewer hyperparameters.

## 2. Related Work

Age estimation is generally considered a regression or classification problem. Earlier studies were based on artificial feature extraction and used various classifiers in machine learning to estimate ages. In classification methods, age estimation is mainly based on these features, and classification [18] adopts support vector machines or random forest and other classifiers. In regression methods, support vector regression (SVR) [19] or partial least squares (PLS) [20] are mainly used to predict age values. Guodong and Guowang [21] adopt biological heuristic model to extract face features. Guo et al. [19] propose to treat age as a pattern subspace and build face images based on age. In Geng's work, face feature vectors are extracted using principal component analysis (PCA), and the extracted feature vectors are combined to describe face age features. Nowadays, more and more people are using deep learning methods, which have greatly improved image classification [11], target detection [22, 23], semantic segmentation [24, 25], time series tasks, and many other visual tasks. Wang [26] used convolutional neural network (CNN) as a feature extractor and then trained SVR to achieve age estimation. Channel attention is of great help in extracting age-related features, such as SENet [27]. Currently ,the latest channel attention networks ,FCAnet, and ECAnet [28, 29], pay more attention to the information interaction between local channels.

Divide-And-Conquer for Age Estimation: Divide-And-Conquer method is to put the problem into smaller subproblems, the smaller the subproblem then built, will have to solve the subproblems to merge; finally it is concluded that, in “mother” problem solution, age estimation regression model has the problem of low accuracy, the traditional research methods the method of the machine. Specifically, the traditional learning method is to manually extract features, input the model and train the model to predict the age value, such as the traditional random forest [30]. Recent studies combine deep learning with random forest (NDF) [16] to obtain higher accuracy and interpretability, and residual random forest is used in [31]. Divide-And-Conquer is similar to mathematical induction. It finds the solution equation formula to solve the problem and then designs the recursive program according to the equation formula [32].

**Figure 2:**
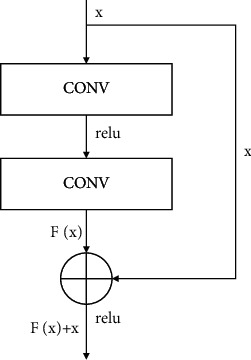
ResNet overview.

Residuals: the residual network unit module contains two convolution layers, as shown in Figure 2. *F* (*x*) is obtained by convolution calculation of input value *X*, and the calculation result of the residual module is obtained by adding *F* (*x*) and *X*, and *F* (*x*) is the residual. Error [33] is mathematically defined as the difference between the observed value and the actual value, while residual is the difference between the predicted value and the observed value. In mathematical statistics, residual is the difference between the actual observed value and the estimated value (fitting value). Through residual, important information about the basic assumptions of the model can be obtained, and the correctness of the regression model can be judged by minimizing residual.

As for the reason for naming the residual network, the author explains that a layer of network can usually be regarded as *y* = *H*(*x*), and a residual network can be expressed as *H*(*x*) = *f*(*x*) + *X*, *f*(*x*) = *H*(*x*) + *X*. In the unit mapping, *y* = *x* is the observed value, but the expected value is *H*(*x*), so it corresponds to the residual *f*(*x*), so it is called the residual network. Short connection: network depth has a great impact on the effect of CNN, but simply increasing network depth cannot simply improve the effect of the network and may destroy the effect of the model due to gradient divergence. The introduction of shortcuts is a way to solve this problem. Expressway is one of the early methods to introduce the idea of shortcut into the depth model, which aims to solve the problems of gradient divergence and difficult training in the depth network. In ResNet, set *t* and *C* of the highway network to 1 to reduce the degree of freedom of the model (in the depth model, the greater the degree of freedom is not necessarily the better). The greater the degree of freedom, the more difficult it is to train. Shortcuts are not limited to one layer, but can also span two or three layers.

Deep forest is a comprehensive forest model [34], which is the integration of traditional forest models in breadth and depth. Although the actual operation occupies more memory and the effect is not as good as deep learning, it also provides an integrated idea for traditional machine learning. Deep forest is made up of different kinds of forests stacked in width and depth. The author Hou [35] has always believed that only by fully reflecting the differences of learning samples can we improve the learning effect of comprehensive learners. Therefore, the stack of deep forest has two purposes: one is to reflect the difference of input data, and the other is to improve the classification ability of input data. The former is called multigranularity scanning and the latter is called cascade forest.

Group Convolution: group convolution was first proposed in Alexnet [11]. Since the network has two GPUs in the experiment, the author hopes to propose packet convolution by paralleling the two models. Channel convolution refers to the characteristic channel. The number of group convolutions of channel convolution is determined by the number of channels in each group. Channel convolution [36] is a part of separable convolution. It is an effective method to improve the recognition accuracy [11] by training a group of independently trained networks and training the grouped channels separately by bisecting the channels. In [37], packet convolution is not trained separately, but jointly, but this simple aggregation does not take into account the information interaction between channels in the channel level convolution channel. The information interaction of multichannel convolution [38] is shown in the figure below. Each convolution core is applied to the input channel of the previous layer to generate an output channel, which is a convolution core of the process. We convolute all channels, repeat the process to generate multiple groups of channels, and then add each channel together to form a final single channel.

## 3. Proposed Method

In this paper, we proposed regression tree age estimation based on local channel attention (CA-XTree), and we will introduce it in detail. ResNet network structure is introduced in feature extraction to help optimize decision function. ResNet improved model (CA-XTree) based on regression tree is composed of two parts: one is ResNet network combined with local channel attention mechanism, and the other is regression tree. The network extracted features through ResNet network added one-dimensional convolution module to extract local channel attention, and input the output results into Softmax function to calculate the final recommendation score *S*.

**Figure 3:**
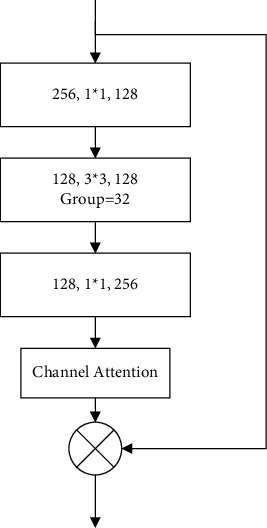
Local channel attention module diagram.

The block convolution module is shown in Figure 3. With a small number of parameters and computation, block convolution can generate a large number of feature graphs and obtain more coded information.

**Figure 4:**
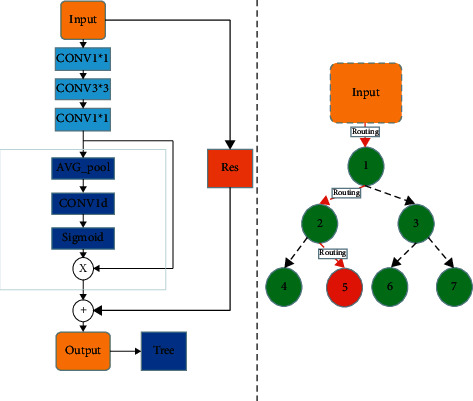
CA-XTree structure. It has two modules: one is local channel attention, and the other is tree classification.

Figure 4 shows the CA-XTree structure. The network extracts feature through ResNet network, adds one-dimensional convolution module to extract local channel attention, and inputs the output results into Softmax function to calculate the final recommendation score S.

### 3.1. Group Convolution

Group convolutions are adopted in the network in this paper. Grouping convolution is used to improve CNN architecture, in which high-dimensional (low-dimensional) channels include long (short) convolution with a fixed number of packets. In other words, the channel dimension C is proportional to the convolution kernel size *K*.

Assume that the input *χ*=[*x*_1_, *x*_2_,…, *x*_*n*_],  *n* ∈ *R*, and we present aggregated transformations as

(1) $$\begin{array}{c} F\left(x\right)=\sum_{i=1}^{j}H_{i}\left(x\right), \end{array}$$

where *H*_*i*_(*x*) is the neuron function, for one neuron, projects *X* into an (optional low dimensional) embed, and then transforms it. In (1), *j* is the size of the set of conversions required for aggregation. In this paper, we design a simple method of transformation function; all *H*_*i*_(*x*) have the same topological structure, with (2) in the aggregate transformation into the residual function:

(2) $$\begin{array}{c} Y=x+\sum_{i=1}^{j}H_{i}\left(x\right). \end{array}$$

### 3.2. Local Cross-Channel Interaction

Let the output of a convolution block be *χ* ∈ *R*^*W*×*H*×*C*^ where *W*, *H*, and *C* are width, height, and channel dimensions as shown in (4), given the aggregated feature.

**Figure 5:**
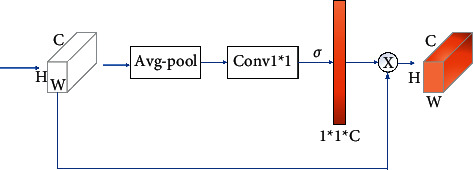
Channel-level attention map.

As shown in Figure 5, the network diagram consists of four parts. Firstly, the aggregation characteristics obtained by global average pool (GAP) are used. Then, the size *k* = 3 is subjected to fast 1D convolution to generate the channel weight, which is multiplied by the feature map to obtain the final result.

*y* ∈ *R*^*C*^ without dimensionality reduction; channel attention can be learned by

(3) $$\begin{array}{c} \omega =\sigma \left(Wy\right). \end{array}$$

*W* is the *C* × *C* parameter matrix, extended into a block diagonal matrix. Divide the channels into *G* groups, each group containing *C*/*G* channels. Channel attention in each group is learned independently to capture cross-channel interactions in a local form:

(4) $$\begin{array}{c} \left[\begin{array}{cccc} \omega^{1,1} & \ldots & \omega^{1,k} & 0\\ 0 & \omega^{2,2} & \ldots & \omega^{2,k+1}\\ \vdots & \vdots & \vdots & \vdots \\ 0 & \ldots & 0 & 0 \end{array}\;\begin{array}{cccc} 0 & \ldots & \ldots & 0\\ 0 & \ldots & \ldots & 0\\ \ddots & \vdots & \vdots & \vdots \\ \ldots & \omega^{C,C-k-1} & \ldots & \omega^{C,C} \end{array}\right]. \end{array}$$

Global average pooling is to output a value by global average of the feature graph, that is, to transform a tensor of *W* × *H*∗ × D into a tensor of 1 × 1 × D. The global average pooling operation is carried out so that it has global receptive field and the global information can be used by the lower layer of the network. Global average pooling (GAP) reduces the number of parameters and can reduce the occurrence of overfitting.

This strategy can be implemented by a one-dimensional fast convolution with a kernel size of *k*:

(5) $$\begin{array}{c} \omega =\sigma \left(C1D_{k}\left(y\right)\right), \end{array}$$

where C1D represents one-dimensional convolution, where the method in (5) is called the CA module, which involves only *k* parameters. Then, given the channel dimension *C*, the kernel size *k* can be adaptively determined:

(6) $$\begin{array}{c} k=\psi \left(C\right)\\ ={\left|\frac{\log_{2}\left(C\right)}{\gamma }+\frac{b}{\gamma }\right|}_{\text{odd}}. \end{array}$$

### 3.3. Neural Decision Forest

Deep neural decision tree (NDF) is a group of deep neural decision trees. For simplicity, each tree is designated as a complete binary tree. We use the integer *I* as the order node.

**Figure 6:**
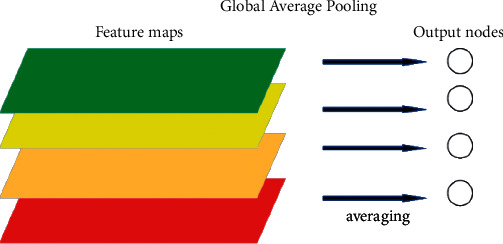
The picture is a global average pooling flowchart.

As shown in Figure 6, the feature map of each channel goes through the global average pooling operation, and the result of this operation is the summary of the global information of each channel, which is calculated by the sigmoid function. The result calculated by sigmoid function is input into node Ri, and depth features are extracted from the input combined with the associated separation node Si, and a recommendation score (routing probability) is given. According to the score value, if it is equal to zero, it is input into the left subtree; if it is equal to 1 it is input into the right subtree. We calculate a unique path from the root to the leaf. In order to obtain the final prediction result, each leaf node contributes its prediction vector according to the probability weight of its calculated path:

(7) $$\begin{array}{c} P=\sum_{i\in N_{l}}\omega_{i}p_{i}. \end{array}$$

The weight of the leaf node is calculated by iteratively adding the weight score of the previous node multiplied by the recommended score. Assuming that the leaf node is on the left subtree of a path, the weight can be expressed as

(8) $$\begin{array}{c} \omega_{i}=\prod_{m=1}^{n}{\left(s_{i_{m}}\right)}^{D\left(j_{m}=0\right)}{\left(1-s_{i_{m}}\right)}^{D\left(j_{m}=1\right)}, \end{array}$$

where it is assumed that the input left node on the path *j*_*m*_=0; otherwise *j*_*m*_=1. Then the weight can be expressed as follows.

If the current formula is valid and the sum of the weights of all leaves is set as 1, it is possible that the final predicted value is a convex function composed of all leaf nodes whose function value converges to 1. Let the above functions be differentiable. Therefore, the loss function defined on the final prediction is made by gradient descent method, and the network weight is updated and the model is trained by minimizing the loss function. We use ResNet network to extract features from inputs, assign values to channels through local channel attention module, input scoring function, combine deep network and regression tree, assign each split node to a neuron in the final full connection layer, and calculate the final recommendation score using S-shaped function. The details are as follows:

(9) $$\begin{array}{c} S_{i}=R_{i}\left(x\right)\\ =\sigma \left(f_{i}\left(M_{n}\left(\ldots M_{1}\right)\right)\right), \end{array}$$

where *M*_*l*_ is the kth feature mapping function represented by one or more layers in the deep neural network, and *f*_*i*_ is a linear mapping function related to the neurons allocated in the last full connection layer, wherein we specify the feature mapping function as

(10) $$\begin{array}{c} M_{k}\left(x\right)=x+H_{k}\left(x\right). \end{array}$$

Since this problem is a multitask regression task, it has *D*=(*x*_*i*_, *y*_*i*_)_*i*_^*N*^=1 instances; this paper directly uses the square loss function:

(11) $$\begin{array}{c} L\left(D\right)=\frac{1}{2}\sum_{i=1}^{N}{\left\|P_{i}-y_{i}\right\|}^{2}. \end{array}$$

The squared loss function means that the output of the model is a Gaussian distribution with the predicted value as the mean value, the loss function is the likelihood of the real value under this predicted distribution, and the Softmax loss means the likelihood of the real tag.

## 4. Experiments

The research object of this paper is static face image, and individual aging is a slow and gradual process; because the gender, race, and different people will be different in aging rate, aging characterization is not the same.

### 4.1. Dataset

For the extraction of age features, due to the size of the finite receptive field and the interaction between cross-channels, the sample feature extraction has a great difference. Therefore, this paper adopts OpenCV and Dlib for face detection and alignment and adopts the method based on channel attention to weaken the cross-channel information interaction in feature extraction. In this paper, face markers are used to locate face regions and eliminate intraface rotation. OpenCV and Dlib are used for face detection and comparison. Finally, resize all images to 256 × 256 pixels, normalize the image according to the calculated mean and standard deviation of the three-color channels, and finally input the model. The training data is expanded by horizontal flipping and random cutting, and the final input space is 224 × 224 pixels.

**Figure 7:**
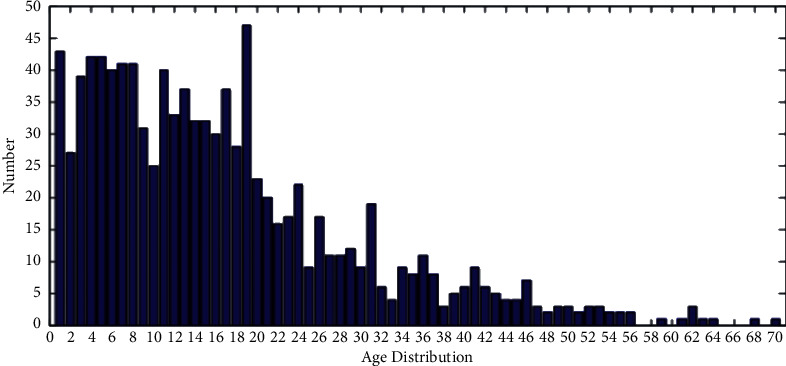
FGNET dataset.

FGNET: the dataset FGNET [39] is shown in Figure 7. The dataset contains 1002 face images of 82 subjects, and 68 faces in each face image in the dataset have manually labeled key points. FGNET dataset has been used in many studies, such as age estimation, cross-age face recognition, age change inference, and other directions [16, 40].

**Figure 8:**
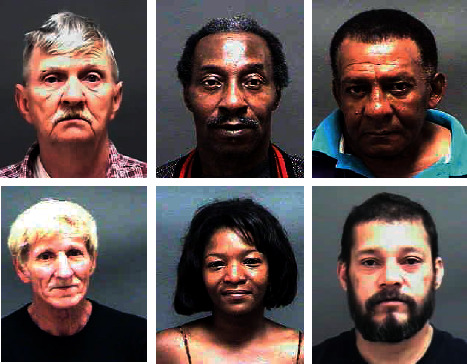
MORPH dataset.

MORPH: the MORPH of the dataset [41] is shown in Figure 8. The dataset contains 13,000 images of faces of different races, skin colors, and genders, of which 55,134 were manually tagged. On the basis of the experiment [16], we select similar test set and training set selection method, randomly select 80% as training set and 20% as test set, and verify the image.

**Figure 9:**
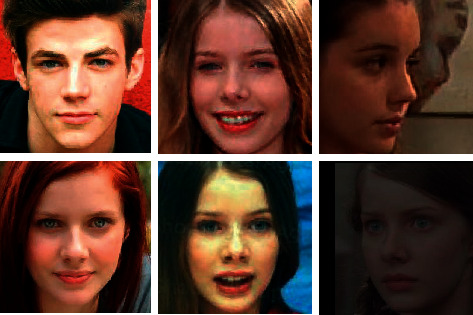
CACD dataset.

CACD: the dataset CACD [1] is shown in Figure 9. The dataset is a 16 GB dataset containing 166,417 photos collected from the Internet, mostly celebrity images. In this paper, the dataset is divided into three sets, namely, the training set, the test set, and the verification set: the training set contains 145,275 images of 1800 celebrities, the test set contains 10,517 images of 120 celebrities, and the verification set contains the remaining 80 celebrities. We train our models through training sets and report on their performance through self-testing.

**Figure 10:**
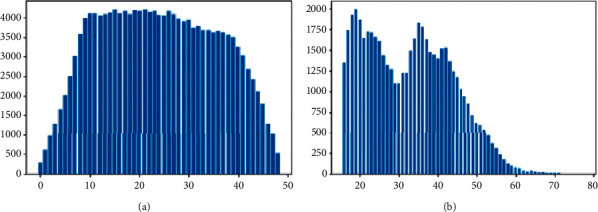
Histogram of (a) CACD datasets (b) MORPH and.

Figure 10 shows the iteration of the training model in the CACD dataset after adding the local channel attention module using the same backbone network. On the premise of introducing block convolution, the experimental results are compared.

The experiment in this paper was conducted on FGNET, MORPH, and CACD datasets. The training set and test set of the dataset are shown in Table 1.

Evaluation criteria: on CACD, FGNET, and MORPH2 datasets, we used the mean absolute error (MAE) and cumulative score (CA) to evaluate the performance. MAE and CS reflect the performance superiority of this model.

(12) $$\begin{array}{c} MAE=\frac{1}{N}\sum_{i=1}^{N}\left|f\left(x_{i}\right)-y_{i}\right|,\\ CA\left(0\right)=\frac{1}{N}\sum_{i=1}^{N}1\left|f\left(x_{i}\right)-y_{i}\right|\leq \theta , \end{array}$$

where *f*(*x*_*i*_) is the predicted output of the network model, *y*_*i*_ is the label corresponding to the input of the face image, and *θ* is the allowable age error corresponding to the cumulative accuracy.

The results show that when the grouping cardinality = 16, the result is the best. This method can effectively improve the age recognition ability of the network model, generate continuous information in the adjacent age range, and improve the generalization ability of the model.

As shown in Table 2, on FGNET and CACD datasets, ResNet-50 was used as the backbone network to test the influence of different grouping numbers on the experimental results.

**Figure 11:**
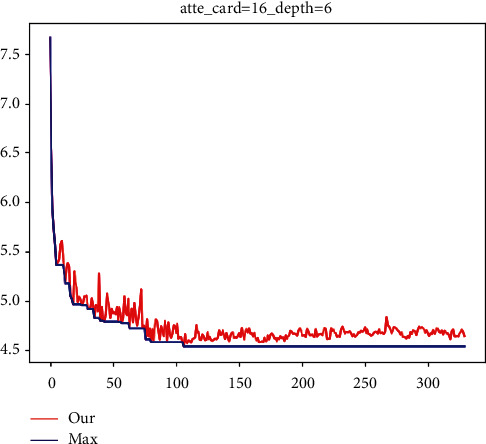
MAE curve chart.

Gradient regression tree: see Figure 11; the local attention module is added, the cardinality is equal to 16, and MAE value is 4.535 which is obtained by tree classification method.

**Figure 12:**
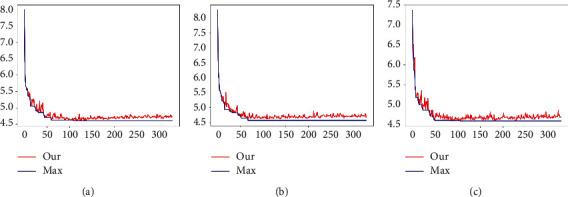
CA-XTree training. Atte represents local channel attention; card represents group number, which is divided into 8, 16, and 32 on the way. (a) atte_card = 8. (b) atte_card = 16. (c) atte_card = 32.

Combine local concerns and grouping convolution as shown in Figure 12. When group *C* is 8, 16, and 32, MAE is 4.6010, 4.567, and 4.6011, respectively. It can be seen from the figure above that *C* = 8, 16 training converges. The information between age features is simply extracted through local channel attention. Then ResNet is introduced into the regression tree by using a simple sigmoid function. Finally, the final result is trained by the regression tree. MAE reached 4.535, which is worthy of further use in future studies. As shown in Table 3, on FGNET and CACD datasets, ResNet-50 as the backbone network and the addition of local channel attention module significantly improved the CACD dataset, MAE value increased by 0.12, and the CS(3) increased by about 1.01%.

All methods extract depth features from the backbone network and then train the generated network in different modules. In this paper, local attention module and grouping volume are combined, and decision tree is used as classification method for training. The experimental results show that 16 groups of 3-tree depth networks have achieved good training results.

### 4.2. Implementation Details

Preprocessing: we use OpenCV and Dlib for face detection and alignment. We adjusted the size of all the images to 256 × 256; then, the image is normalized according to the calculated mean and standard deviation of the three-color channel, and the training data is expanded by 0.5 probability level flipping and random clipping. Finally, the input image size is 224 × 224.

Model architecture: we select ResNet-50 as the baseline network, add the aggregation attention module, and then use two full connection layers. The final output is activated by sigmoid function and sent to the decision forest to give the final result. Hyperparameter setting: in order to compare with the previous work, we used a forest of 5 trees, each with a depth of 6. Another forest with a depth of 7 was also used. The batch is 64, which is used for back propagation to train network parameters. We add a new leaf node prediction vector to every 50 batches of network parameters and randomly take 500 samples each time.

Training settings: with the SGD optimizer, the initial learning rate was set to 0.5, and when training with the scheduler model provided by PyTorch stalled, the learning rate was halved.

Table 4 shows the experimental results of the CACD dataset. All methods extract depth features from the backbone network and then train the generated network in different modules. In this paper, the local attention module and grouping convolution are combined, and the depth of the tree is tested. The experimental results show that, in ResNet-50 network as the backbone network, the grouping number is *C* = 16 and MAE value reaches 4.535, which is about 0.135 higher than that of the latest method. CS(3) cumulative score reaches 63.53%, which is about 2.13% higher than that of the latest method.

In the experiment, the tree depth was studied. When the depth was 8, the loss of computing resources was too large; when the depth was 5, the tree leaf node was less than 32, affecting the accuracy of age estimation.  Table 5 shows the experimental results on dataset CACD when the tree depth is 7.

**Figure 13:**
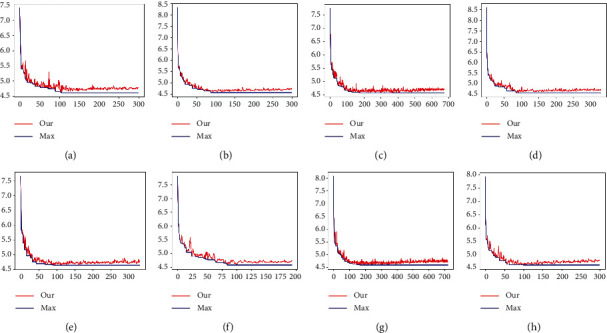
Results. Similar fluctuation of network training and its corresponding network training iteration results. When group number C is 2, 4, 8, and 32 and tree depth is 6, MAE is 4.612, 4.561, 4.569, and 4.579, respectively. Group C is 2, 4, 8, and 32 and tree depth is 7. (a) atte_card = 2_depth = 6. (b) atte_card = 4_depth = 6. (c) atte_card = 8_depth = 6. (d) atte_card = 32_depth = 6. (e) atte_card = 2_depth = 7. (f) atte_card = 4_depth = 7. (g) atte_card = 8_depth = 7. (h) atte_card = 32_depth = 7.

Figure 13 shows the variation curve of the error over time. As shown in Figure 13, the final experimental performance reaches 4,553.

In this paper, the size *k* of convolution kernel is adaptively determined by using the global average pooling aggregation of convolution features without dimensionality reduction, and then convolution operation is carried out and sigmoid function is executed to learn channel attention. This paper applies the attention module to the deep convolution neural network based on ResNet-50. This paper tests FGNET dataset and CACD dataset. The mean absolute error (MAE) of CACD was 4.567 and the cumulative score was 61.11%, and the mean absolute error (MAE) of dataset FGNET is 2.953 and the cumulative score is 83.8%.

Table 6 shows the comparison results of CA-XTree with the current advanced methods, with different results on different datasets. In the FGNET dataset, the dataset is 2004, the data image clarity is insufficient, and the accuracy is slightly reduced; CACD dataset was proposed in 2016, and the size was about 14G. Experimental results were obtained on CACD, which was relatively ideal. Compared with the 2021 CORF method, it achieved an improvement of 0.135 in large dataset CACD.

## 5. Conclusion

In this paper, we use the residual network as the backbone network to repeatedly establish a building block, the aggregation of building blocks, a set of the same topological transformation, and the attention of a channel, do not reduce the dimension module, and simply set some parameters, and the number and memory loss do not increase too much. Finally, we deeply study the soft decision function of neural decision forest. See Table 6 for model accuracy and compare it with previous work. Our model achieves the highest accuracy in all benchmarks and achieves significantly better performance on the largest dataset CACD. The age estimation dataset of CA-XTree network is mainly composed of two-dimensional face images, while outdoor video surveillance is a video of face. The next work uses a multiview spatial attention mechanism to extract and integrate multiview features, so as to better extract local information of age features. Then two fully connected layers were used for age estimation.

## Figures and Tables

**Table 1:** Dataset training set and test set.

Datasets	Instances	Training	Testing	Age range
MORPH	55000	44000	11000	16–77
FGNET	1002	800	200	0–69
CACD	166417	145275	10571	16–62

**Table 2:** Ablation experiments on CACD.

Baseline	Setting	MAE (FGNET)	MAE (CACD)	CS(3) (CACD) (%)
ResNet-50	*c* = 2	2.95	4.63	61.84
ResNet-50	*c* = 4	3.01	4.64	61.58
ResNet-50	*c* = 8	2.91	4.61	61.14
ResNet-50	*c* = 16	2.94	4.67	61.34
ResNet-50	*c* = 32	2.99	4.71	61.68

“c” indicates the number of groups.

**Table 3:** The comparison results of CACD datasets.

Baseline	Setting	MAE (FGNET)	MAE (CACD)	CS(3) (CACD) (%)
ResNet-50	*c* = 2_CA	2.79	4.593	62.06
ResNet-50	*c* = 4_CA	2.83	4.561	62.28
ResNet-50	*c* = 8_CA	2.70	4.575	62.85
ResNet-50	*c* = 16_CA	2.69	4.553	62.20
ResNet-50	*c* = 32_CA	2.73	4.61	62.61

**Table 4:** Ablation experiments on CACD. CA is channel attention.

Baseline	Setting	MAE (CACD)	CS(3) (CACD) (%)
ResNet-50	*c* = 2_CA_tree = 6	4.590	62.84
ResNet-50	*c* = 4_CA_tree = 6	4.561	63.53
ResNet-50	*c* = 8_CA_tree = 6	4.569	63.26
ResNet-50	*c* = 16_CA_tree = 6	4.535	62.26
ResNet-50	*c* = 32_CA_tree = 6	4.579	63.37

CA is channel attention. “c” indicates the number of groups.

**Table 5:** Ablation experiments on CACD. CA is channel attention.

Baseline	Setting	MAE (CACD)	CS(3) (CACD) (%)
ResNet-50	*c* = 2_CA_tree = 7	4.639	62.50
ResNet-50	*c* = 4_CA_tree = 7	4.578	63.04
ResNet-50	*c* = 8_CA_tree = 7	4.593	62.58
ResNet-50	*c* = 16_CA_tree = 7	4.575	62.23
ResNet-50	*c* = 32_CA_tree = 7	4.598	62.48

“c” indicates the number of groups. CA stands for local channel attention.

**Table 6:** Mean absolute error (MAE) of different methods.

Method	Year	FGNET	CACD
DIF	2015	4.80/74.3%	—/—
Human workers	2015	4.70/69.5%	—/—
DLA	2015	4.26/—	—/—
DEX [34]	2016	4.63/—	4.785/—
dLDLF [42]	2017	—/—	4.734/—
DRFs [16]	2018	3.85/80.6%	4.637/—
RNDF [31]	2019	3.87/76.1%	4.595/—
CORF [17]	2021	2.68/86.80%	4.67
CA-XTree	2021	2.69	4.554

## Data Availability

Contact the authors for the data used in this study if necessary.

## References

[1] Chen B.-C., Chen C.-S., Hsu W. H. (2015). Face recognition and retrieval using cross-age reference coding with cross-age celebrity dataset. *IEEE Transactions on Multimedia*.

[2] Liu H., Lu J., Feng J., Zhou J. (2017). “Label-sensitive deep metric learning for facial age estimation. *IEEE Transactions on Information Forensics and Security*.

[3] Liu N., Zhang F., Duan F. (2020). Facial age estimation using a multi-task network combining classification and regression. *IEEE Access*.

[4] Li P., Hu Y., Li Q., He R., Sun Z. Global and Local Consistent Age Generative Adversarial Networks.

[5] Chang K.-Y., Chen C.-S., Hung Y.-P. Ordinal Hyperplanes Ranker with Cost Sensitivities for Age Estimation.

[6] Yi J., Mao X., Chen L., Rovetta A. (2016). Illumination compensation for facial feature point localization in a single 2D face image. *Neurocomputing*.

[7] Wang N., Gao X., Tao D., Yang H., Li X. (2018). Facial feature point detection: a comprehensive survey. *Neurocomputing*.

[8] Deng J., Zhou Y., Zafeiriou S. Marginal loss for deep face recognition.

[9] Dornaika F., Bekhouche S., Arganda-Carreras I. (2020). Robust regression with deep CNNs for facial age estimation: an empirical study. *Expert Systems with Applications*.

[10] Xiao C., Zhifeng Z., Jie C., Qian Z. (2021). Combined deep learning with directed acyclic graph SVM for local adjustment of age estimation. *IEEE Access*.

[11] Krizhevsky A., Sutskever I., Hinton G. E. (2012). Imagenet classification with deep convolutional neural networks. *Advances in neural information processing systems*.

[12] Fu J., Liu J., Tian H. Dual attention network for scene segmentation.

[13] Banerjee S. (1997). From the desk of the editor. *Indian Journal of Otolaryngology and Head & Neck Surgery*.

[14] Kontschieder P., Fiterau M., Criminisi A., Bulo S. R. Deep neural decision forests.

[15] Roy A., Todorovic S. Monocular depth estimation using neural regression forest.

[16] Shen W., Guo Y., Wang Y., Zhao K., Wang B., Yuille A. L. Deep regression forests for age estimation.

[17] Zhu H., Shan H., Zhang Y. (2021). Convolutional ordinal regression forest for image ordinal estimation. *IEEE Transactions on Neural Networks and Learning Systems*.

[18] Geng X., Zhou Z.-H., Smith-Miles K. (2007). Automatic age estimation based on facial aging patterns. *IEEE Transactions on Pattern Analysis and Machine Intelligence*.

[19] Guo G., Mu G., Fu Y., Huang T. S. Human Age Estimation Using Bio-Inspired Features.

[20] Guo G., Mu G. Human Age Estimation: What Is the Influence across Race and Gender?.

[21] Guodong G., Guowang M. Simultaneous dimensionality reduction and human age estimation via kernel partial least squares regression.

[22] Girshick R., Donahue J., Darrell T., Malik J. Rich feature hierarchies for accurate object detection and semantic segmentation.

[23] Xia M., Liu W. a., Shi B., Weng L., Liu J. (2019). Cloud/snow recognition for multispectral satellite imagery based on a multidimensional deep residual network. *International Journal of Remote Sensing*.

[24] Long J., Shelhamer E., Darrell T. Fully convolutional networks for semantic segmentation.

[25] Xia M., Qian J., Zhang X., Liu J., Xu Y. (2019). River segmentation based on separable attention residual network. *Journal of Applied Remote Sensing*.

[26] Xia M., Liu W. a., Wang K., Xu Y. (2019). Non-intrusive load disaggregation based on deep dilated residual network. *Electric Power Systems Research*.

[27] Hu J., Shen L., Sun G. Squeeze-and-excitation networks.

[28] Qin Z., Zhang P., Wu F., Li X. Fcanet: frequency Channel Attention networks.

[29] Wang Q., Wu B., Zhu P., Li P., Zuo W., Hu Q. Ecanet: efficient channel attention for deep convolutional neural networks.

[30] Montillo A., Ling H. Age Regression from Faces Using Random Forests.

[31] Li S., Cheng K.-T. (2019). Facial age estimation by deep residual decision making. https://arxiv.org/abs/1908.10737#:%7E:text=Residual%20representation%20learning%20simplifies%20the,neural%20decision%20forest%20.

[32] Hou Q., Zhou D., Feng J. Coordinate attention for efficient mobile network design.

[33] Wc A., Vm B., Sr A. (2020). Rank consistent ordinal regression for neural networks with application to age estimation. *Pattern Recognition Letters*.

[34] Xia M., Zhang X., Liu W. a., Weng L., Xu Y. (2020). Multi-stage feature constraints learning for age estimation. *IEEE Transactions on Information Forensics and Security*.

[35] Zhang X., Wang J., Wang T., Jiang R., Xu J., Zhao L. (2021). Robust feature learning for adversarial defense via hierarchical feature alignment. *Information Sciences*.

[36] Sifre L., Mallat S. (2014). Rigid-motion scattering for texture classification. https://arxiv.org/abs/1403.1687.

[37] Xie S., Girshick R., Dollar P., Tu Z., He K. Aggregated residual transformations for deep neural networks.

[38] Hsu G. S. J., Wu H. Y., Yap M. H. A comprehensive study on loss functions for cross-factor face recognition.

[39] Lanitis A., Taylor C. J., Cootes T. F. (2002). Toward automatic simulation of aging effects on face images. *IEEE Transactions on Pattern Analysis and Machine Intelligence*.

[40] Rothe R., Timofte R., Van Gool L. (2018). Deep expectation of real and apparent age from a single image without facial landmarks. *International Journal of Computer Vision*.

[41] Rawls A. W., Ricanek K. (2009). MORPH: development and optimization of a longitudinal age progression database. *Biometric ID Management and Multimodal Communication*.

[42] Shen W., Zhao K., Guo Y., Yuille A. (2017). Label distribution learning forests. https://arxiv.org/abs/1702.06086.

